# Multimodal tract-based MRI metrics outperform whole brain markers in determining cognitive impact of small vessel disease-related brain injury

**DOI:** 10.1007/s00429-022-02546-2

**Published:** 2022-08-22

**Authors:** Alberto De Luca, Hugo Kuijf, Lieza Exalto, Michel Thiebaut de Schotten, Geert-Jan Biessels, The Utrecht VCI Study Group, E. van den Berg, G. J. Biessels, L. G. Exalto, C. J. M. Frijns, O. Groeneveld, R. Heinen, S. M. Heringa, L. J. Kappelle, Y. D. Reijmer, J. Verwer, N. Vlegels, J. de Bresser, A. De Luca, H. J. Kuijf, A. Leemans, H. L. Koek, M. Hamaker, R. Faaij, M. Pleizier, E. Vriens

**Affiliations:** 1 VCI Group, Neurology Department, UMC Utrecht Brain Center, UMC Utrecht, Utrecht, The Netherlands; 2 Image Sciences Institute, Division Imaging and Oncology, UMC Utrecht, Utrecht, The Netherlands; 3 Brain Connectivity and Behaviour Lab, Sorbonne University, Paris, France; 4 Institut des Maladies Neurodégénératives, Neurofunctional Imaging Group, University of Bordeaux, Bordeaux, France

**Keywords:** Cerebral small vessel disease, Diffusion MRI, Fiber tractography, Cognition, Neural network

## Abstract

In cerebral small vessel disease (cSVD), whole brain MRI markers of cSVD-related brain injury explain limited variance to support individualized prediction. Here, we investigate whether considering abnormalities in brain tracts by integrating multimodal metrics from diffusion MRI (dMRI) and structural MRI (sMRI), can better capture cognitive performance in cSVD patients than established approaches based on whole brain markers. We selected 102 patients (73.7 ± 10.2 years old, 59 males) with MRI-visible SVD lesions and both sMRI and dMRI. Conventional linear models using demographics and established whole brain markers were used as benchmark of predicting individual cognitive scores. Multi-modal metrics of 73 major brain tracts were derived from dMRI and sMRI, and used together with established markers as input of a feed-forward artificial neural network (ANN) to predict individual cognitive scores. A feature selection strategy was implemented to reduce the risk of overfitting. Prediction was performed with leave-one-out cross-validation and evaluated with the R^2^ of the correlation between measured and predicted cognitive scores. Linear models predicted memory and processing speed with *R*^2^ = 0.26 and *R*^2^ = 0.38, respectively. With ANN, feature selection resulted in 13 tract-specific metrics and 5 whole brain markers for predicting processing speed, and 28 tract-specific metrics and 4 whole brain markers for predicting memory. Leave-one-out ANN prediction with the selected features achieved *R*^2^ = 0.49 and *R*^2^ = 0.40 for processing speed and memory, respectively. Our results show proof-of-concept that combining tract-specific multimodal MRI metrics can improve the prediction of cognitive performance in cSVD by leveraging tract-specific multi-modal metrics.

**Supplementary Information:**

The online version contains supplementary material available at 10.1007/s00429-022-02546-2.

## Introduction

Cerebral small vessel disease (cSVD) is a major cause of cognitive decline (Gorelick et al. 2011; Iadecola et al. 2019) and one of the leading causes of dementia (Iadecola 2013), often as a co-morbidity to Alzheimer’s disease. Brain injury in patients with cSVD can be assessed with complementary MRI techniques. Structural MRI (sMRI), such as T1-weighted imaging and fluid attenuated inversion recovery (FLAIR), provides imaging markers of brain atrophy and lesion burden (e.g., volume or count), such as white matter hyper-intensities (WMH), micro-bleeds, and lacunar infarcts (Wardlaw et al. 2013). Diffusion MRI (dMRI) leverages sensitivity to the motion of water molecules at the microscopic scale to detect microstructural tissue alterations in cSVD. Metrics quantified with dMRI, such as the mean diffusivity (MD) or peak-skeletonized mean diffusivity (PSMD) (Baykara et al. 2016), have been shown promising to detect cSVD-related injury beyond visible lesions (Finsterwalder et al. 2020), also in normal appearing brain tissue. The relation between cSVD lesions visible on MRI and cognitive function remains overall poorly understood. While at the population level WMH burden clearly associates with dementia risk (Debette et al. 2019), in individual patients, this relation is variable. This can create diagnostic dilemmas. For example, in a memory clinic setting, a patient with subjective complaints may have the same WMH burden as a patient with cognitive impairment attributed to cSVD. Even in research settings, the actual explained variance in cognition for individual MRI metrics remains modest. For example, a prediction model combining patient demographics, whole brain markers of lesions and atrophy, and PSMD only explained 8–16% of variance in cognitive performance in sporadic cSVD (Baykara et al. 2016). There is therefore a need for tools that can capture more relevant features of MRI detectable abnormalities to better explain the deficit of an individual patient, ultimately supporting diagnosis. Moreover, better understanding the relation between MRI markers and concomitant cognitive function may also provide new insights into the mechanisms through which brain lesions cause cognitive impairment.

An emerging approach to characterize cognitive decline in cSVD is to consider not only injury burden, but also its location on the brain circuitry. For example, damage of specific white matter (WM) tracts in patients with stroke was shown to be linked to impairment of specific brain functions (Rojkova et al. 2016; Howells et al. 2018; Thiebaut de Schotten et al. 2020). Moreover in cSVD it has been shown that diffusion tensor imaging (DTI) metrics of specific WM tracts—either derived with fiber tractography or using standardized atlases—are better predictive of cognitive performance than whole brain DTI metrics (Biesbroek et al. 2018). Hence, a promising way forward to achieve larger sensitivity to outcomes of interest (Zeestraten et al. 2017; de Lange et al. 2020) (e.g., cognition), is to consider a multi-modal analysis where tract-based metrics from dMRI, markers of brain atrophy from sMRI (e. g., cortical thickness), lesion markers (e.g., WMH burden) and clinical covariates (e.g., age, gender, education level) are integrated.

Considering together multiple MRI metrics can be challenging with conventional statistical approaches such as linear models (Chamberland et al. 2019; Muncy et al. 2022)—the leading analysis method in cSVD research. Indeed, metrics derived from the same imaging modality and sampled in different brain regions (e.g., at the tract level) are likely to be collinear, and create instability in regression modeling. Adding large numbers of predictors to the models also puts constrains on statistical power. Furthermore, relations between imaging markers and cognitive performance may be non-linear (Wang et al. 2013; Wan et al. 2014; Cao et al. 2018). A promising way to address these issues is to consider an analysis method able to learn the relation between multiple inputs and outcome in a data-driven fashion. Artificial neural networks (ANN) have recently gained attention as a versatile tool able to map complex relations between imaging metrics and outcome measures in a data-driven fashion, and allow to take into account potential collinearities between imaging metrics, as well as eventual non-linear relations with outcome. Furthermore, their application is supported by high-quality frameworks striving for ease of use and computational performance, which allow to scale up their application on large datasets, an important feature in the upcoming era of big data analysis in SVD (de Luca and Biessels 2021).

In this work, we investigate whether integrating multi-modal metrics of the main WM tracts explains more inter-subject variability in cognitive performance than established whole brain imaging markers of cSVD (Biesbroek et al. 2016, 2017; Boomsma et al. 2020). Our approach includes a fully automated pipeline to derive and integrate established whole brain markers, and sMRI and dMRI metrics sampled both at the whole brain level as well as in 73 WM tracts.

## Methods

### Study sample

The data included in this study include the UMC Utrecht participants of the TRACE-VCI study (Boomsma et al. 2017), a cohort of patients visiting the memory clinic with cognitive complaints and visible vascular lesions on their brain MRI. During their assessment at the memory clinic, participants underwent a 3 Tesla MRI scan with a standardized protocol including T1-weighted imaging with resolution 1 × 1 × 1 mm^3^, a fluid-attenuated inversion recovery (FLAIR) acquisition with resolution 0.96 × 0.96 × 3.00 mm^3^, and a diffusion MRI scan with resolution 2.5 mm^3^ isotropic including 45 gradient directions at *b* = 1200 s/mm^2^ in addition to 1 *b* = 0 s/mm^2^ averaged three times. Next to MRI, all participants underwent a standardized neuropsychological evaluation to assess their cognitive status. ﻿The study was approved by the institutional review board of the UMC Utrecht. All patients provided informed consent prior to research-related procedures.

The severity of WMH burden was rated with the Fazekas score only considering deep and not periventricular lesions, as follows: 0 = absence, 1 = punctate foci, 2 = beginning confluence of foci, 3 = large confluent areas. Out of the 196 available subjects, we selected only patients exhibiting manifestations of cSVD, which was operationalized as having a Fazekas score ≥ 2, or presence of (small) subcortical or lacunar infarcts. Patients were excluded in presence of infarct(s) or hemorrhage(s) with volume above 4.2 mL (i.e., the equivalent of a spherical lesion with a diameter > 2 cm) or incidental findings (i.e., brain cancer, cysts) on MRI affecting analyses. This arbitrary volume cut-off was primarily used because larger lesions by themselves more likely affect cognition.

Of the 116 subjects selected with the abovementioned criteria, 14 were further discarded because of incomplete cognitive assessment (*n* = 11) or poor MRI quality (*n* = 3), resulting in the final selection of 102 subjects reported in Table 1. A flow chart summarizing our inclusion and exclusion criteria is shown in the Supplementary Material, Figure S1.

**Table 1:** Demographic characteristics, clinical diagnosis, and cognitive evaluation of the study participants

Included patients *n* = 102	
Demographics	
Sex, % men	#59 males (58%)
Age (years)	73.7 (67.4–81.7)
Level of education^a^	5 (4–6)
Clinical diagnosis	
No objective cognitive impairment	#18 (18%)
MCI	#31 (30%)
Dementia	#53 (52%)
Vascular dementia	#6 (6%)
Alzheimer’s disease	#41 (40%)
Other neurodegenerative etiology	#5 (5%)
Unknown etiology	#1 (1%)
Measures of global cognitive status	
Mini-mental state examination	26.5 (24–28)
Clinical dementia rating	0.5 (0.5–1)
Cognitive performance	
Processing speed	0.09 (− 0.4 to 0.5)
Memory	− 0.15 (− 0.8 to 0.3)
^a^Verhage scale: low education (1–4), middle education (5), high education (6–7)	

Numbers before brackets indicate either the count (#) or the median. Numbers between brackets indicate the 25th and 75th percentile, or the percentage (%)

### Cognitive performance

A detailed explanation of the cognitive evaluation of the study sample can be found in a previous work (Boomsma et al. 2017). In short, level of education was defined according to a 7-point rating scale [Verhage scale (Verhage 1964) 1–7; low to high education]. Cognition was first screened with the Dutch version of the Mini Mental State Examination (MMSE, max. score 30). The severity of cognitive symptoms was assessed with the Clinic Dementia Rating score (CDR, 0–3). Patients received a multidomain cognitive assessment. For the present study, we considered the domains memory and processing speed.

The domain memory was assessed by the Dutch version of the Rey Auditory Verbal Learning Test (RAVLT). For the RAVLT, the total number of words remembered in five learning trials was recorded and the delayed recall and recognition tasks were used. Furthermore, the Visual Association Test (VAT) part A was included to assess visuospatial association learning.

The domain information processing speed was assessed by the Trail Making Test Part A (TMTA-A), the Stroop Color Word Test I and II, and the Digit Symbol-Coding Test (DSCT) of the WAIS-III or the Letter Digit Substitution Test (LDST). Z-scores were created for each individual test (reversed *Z*-scores for the TMT and Stroop Color Word Test).

Individual test scores of all subjects were transformed to z-scores, e.g., subtracting the average and dividing by the standard deviation of all subjects, then averaged to create domain *Z*-scores. Accordingly, a *z*-score equal to 0 indicates the average cognitive value in the whole cohort, and not an intact average cognitive score.

Participants were clinically classified as follows:

- No objective cognitive impairment, when having cognitive complaints but no objective impairment on neuropsychological testing.
- Mild cognitive impairment (MCI), when observing deterioration in cognitive function as compared to a previous time point, and objective impairment in at least one cognitive domain.
- Dementia, when observing objective impairment in two or more cognitive domains. Dementia was further classified based on its main etiology using internationally established criteria in the following subtypes: vascular (Roman et al. 1993), Alzheimer’s disease (McKhann et al. 1984), other neurodegenerative etiology (McKeith et al. 2005; Rascovsky et al. 2011), or unknown origin.

### Data processing

MRI data were processed with an automated pipeline based on CAT12 (http://www.neuro.uni-jena.de/cat/), ExploreDTI (Leemans et al. 2009) and the in-house developed toolbox “MRIToolkit” (Guo et al. 2020) (https://github.com/delucaal/MRIToolkit).

T1-weighted and FLAIR images were processed with CAT12 to derive automatic segmentations of white matter, gray matter, cerebrospinal fluid and white matter hyper-intensities (Tohka et al. 2004). All segmentations were individually inspected to ensure they were of sufficient quality and did not contain major errors. Additionally, the cortical thickness (CTH) (Yotter et al. 2011; Dahnke et al. 2013) was evaluated. Next, micro-bleeds, infarcts, and hemorrhages were evaluated by a trainer rater using the FLAIR images as previously described (Boomsma et al. 2020).

dMRI data were corrected for signal drift (Vos et al. 2016) and Gibbs’ ringing (Perrone et al. 2015), then motion, Eddy currents and echo-planar-imaging (EPI) corrections with b-matrix rotation were performed in one step. The latter step was performed using the T1-weighted image resampled to 2 × 2 × 2 mm^3^ as target. Next, a robust fit of the diffusion tensor was performed with REKINDLE (Tax et al. 2015). Visual inspection was performed to effectiveness of motion correction and registration to the T1-weighted image, as well as the presence of major data artifacts in the DTI fit residuals.

Constrained spherical deconvolution (CSD) (Tournier et al. 2007) was performed using spherical harmonics of order 6 and recursive calibration of the response function (Tax et al. 2014) to determine the fiber orientation distribution, then deterministic fiber tractography was applied using each brain voxel as a seed, with angle threshold 30°, step size 1 mm. Streamlines shorted than 30 mm or longer than 500 mm were discarded (default values in ExploreDTI). Subsequently, the white matter analysis clustering approach (Zhang et al. 2018) was applied to automatically reconstruct 73 brain tracts based on known anatomy. A list of the reconstructed tracts and their abbreviation is reported in Supplementary Material Table S1. Spatial probability maps of the reconstructed tracts in the whole dataset are reported in Supplementary Information Videos S1–S3.

### Study design

First, we aimed to characterize the maximum amount of variance that conventional linear models can explain at the group level. To this end, we used linear regression to characterize the amount of variance in cognitive scores (i.e., information processing speed, memory performance) explained by models of increasing complexity considering (1) demographics only (i.e., age, sex, level of education); (2) demographics + whole brain markers from sMRI (i.e., WMH burden, brain parenchymal fraction (BPF), presence of lacunes, presence of micro-bleeds); (3) model 2 + the average value of a diffusion metric (i.e., FA or MD or PSMD) in the whole WM. In this analysis, all subjects were used simultaneously (*N* = 102).

Subsequently, we evaluated two methods to predict individualized cognitive function using a leave-one-out validation strategy, which is an arguably harder task than regression and allows to evaluate the generalizability of a prediction model to unseen data. This implies that the prediction was performed 102 times, removing 1 subject each time and re-training the prediction on the remaining 101 subjects. Furthermore, in the supporting information we further evaluated the generalizability of the method by repeating the prediction with a leave-5-out cross-validation scheme.

The first prediction method is based on linear models —the current standard in cSVD— and serves as prediction benchmark. The same metrics considered for linear regression (demographics + whole brain markers from sMRI and dMRI) were considered as input for this prediction strategy. For each input metric, we evaluated its standardized coefficient, its significance (*p*-value) and the amount of variance it explained as quantified by the *R*-squared (*R*^2^). In the supporting information, we also report an evaluation of the prediction performance of linear models when considering tract-based metrics as input.

The second prediction strategy is based on a novel tract-based ANN to predict individualized cognitive function, and is presented in the following section. To compare our proposed strategy to the benchmark, we evaluated the mean absolute error (MAE) of the prediction and its *R*^2^ value. To assess whether tract-based ANN significantly predicted cognitive performance better than conventional models, *F*-tests were performed. Because conventional *F*-tests weight the residuals sum of squares (RSS) by the number of parameters, they are unsuited for evaluating ANNs because of their large number of parameters. Accordingly, we applied a modified *F*-test considering the number of input predictors #*K* in place of the number of parameters, as follows:

$$F = {\raise0.7ex\hbox{${\frac{{{\text{RSS}}_{{{\text{linear}}}} - {\text{RSS}}_{{{\text{ANN}}}} }}{{\# K_{{{\text{ANN}}}} - \# K_{{{\text{linear}}}} }}}$} \!\mathord{\left/ {\vphantom {{\frac{{{\text{RSS}}_{{{\text{linear}}}} - {\text{RSS}}_{{{\text{ANN}}}} }}{{\# K_{{{\text{ANN}}}} - \# K_{{{\text{linear}}}} }}} {\frac{{{\text{RSS}}_{{{\text{ANN}}}} }}{{N_{{\text{S}}} - \# K_{{{\text{ANN}}}} }},}}}\right.\kern-\nulldelimiterspace} \!\lower0.7ex\hbox{${\frac{{{\text{RSS}}_{{{\text{ANN}}}} }}{{N_{{\text{S}}} - \# K_{{{\text{ANN}}}} }},}$}}$$

where *N*_S_ is the number of subjects (102).

### Tract-based ANN prediction

#### ANN features sampling

**Fig. 1:**
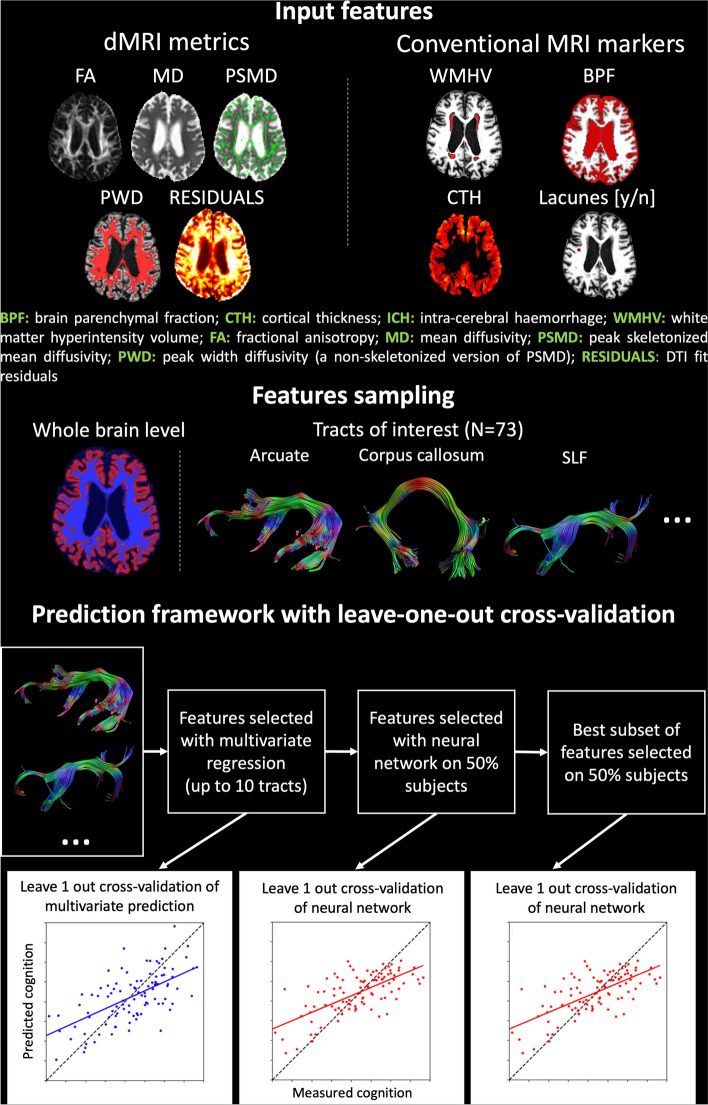
An overview of the framework used in this work. Multi-modal metrics computed from the diffusion tensor (FA, MD, PSMD, RESIDUALS), T1-weighted imaging (CTH) and FLAIR (WMH) are derived at (i) the whole brain level and ii) for each major white matter tracts of the 73 obtained with an automatic tractography clustering method. The considered measures are used as input to a linear multivariate prediction model and an artificial neural network (ANN) with leave-one-out cross-validation

In our prediction framework, we integrated multi-modal MRI metrics sampled both at the whole brain level and in 73 WM tracts, as depicted in Fig. 1. At the whole brain level, we considered the same markers used as input for linear prediction. Additionally, we considered the average CTH, and the mean squared error of the DTI fit residuals, which informs on both data quality and appropriateness of the model. Residuals assume high values in presence of outliers in the data, but also in case of non-Gaussian diffusion effects in the data (van Rijn et al. 2020) owing to, for example, microstructural alterations (Jensen et al. 2005; Goghari et al. 2021).

To extract tract-specific metrics, the volumetric representation of the streamlines of each white matter tract was derived and used as a region of interest. For each tract, we computed the average FA, MD, WMH burden volume (WMHV) and DTI fit residuals without distinguishing between WM and GM. Next, we evaluated the peak width of mean Diffusivity (PWD) for each tract in analogy to the whole brain PSMD, e.g., by determining the difference between the 75th and 25th percentile of MD within each tract mask. Additionally, the average CTH of each tract was calculated as the average thickness of the cortex adjacent to a WM tract.

All metrics were transformed to Z-scores as common practice in machine learning (More et al. 2021) to optimize their use as predictors in subsequent analyses.

#### ANN architecture

Our ANN framework consists of a feed-forward network with 20 nodes and 1 hidden layer (empirically chosen). The input of each layer was normalized (“*BatchNorm1d*”), and the non-linear rectified linear unit function (ReLU) was included as activation function between each layer. The network was implemented in Python using the PyTorch library and trained with the ADAM optimizer using the mean squared error cost function with L1 penalty (“*L1Lasso*”). The learning rate was empirically set to 0.01 after experimentation in the range 0.0001–0.1, and a dropout rate equal to 30% was used. The training dataset (*N* = 101) was split in a training (90%) and validation set (10%) to implement an early stopping strategy, e.g., to interrupt the training once the error in the validation set increases during training. The minimum number of training epochs was 30, and the maximum 300. For each subject, the training and prediction were repeated 30 times to account for non-deterministic processes in ANNs, then the median of all predicted values was taken as final prediction.

#### ANN features selection

We designed a feature selection strategy based to integrate multimodal MRI metrics and predict cognitive performance while minimizing potential risks of over-fit. An overview of our strategy is presented in Fig. 1.

To reduce the number of metrics to be considered for ANN feature selection, we implemented a first filtering step based on linear prediction. To this end, we repeatedly performed a leave-one-out prediction using a single metric (WMHV, FA, MD, PWD, residuals, CTH) sampled for all 73 WM tracts. For each metric, the 7 most significant tracts (e.g., 10% of the total) and their contralateral pathways were selected for the next phase. For the prediction of memory, the superior longitudinal fasciculus and the frontal-thalamic projections were additionally included if not selected at the previous stage, given their previously reported relevance in memory-related tasks (Bolkan et al. 2017; Biesbroek et al. 2018).

Once a set of candidate tracts was determined, these were given as input to an iterative ANN optimization procedure repeated 10 times on random subsets of 51 subjects (50%). The procedure determined the optimal combination of features to predict cognition in the given random subset with a bottom-up strategy. At the first iteration, age and education are the only predictors. Subsequently, the procedure evaluates which of the available metrics improves the prediction performance (*R*^2^) in the random subset and adds it to the predictors list. Given the aleatory nature of ANN, each prediction was repeated three times and the average prediction considered as outcome. The procedure continued until the prediction performance did not further improve.

The feature selection procedure was repeated 10 times to obtain the candidate predictors. We then evaluated the final performance of the ANN at predicting processing speed and memory performance in the complete dataset using (1) the features corresponding to the feature selection iteration achieving the highest *R*^2^, and (2) all candidate predictors determined in the 10 repetitions.

## Results

### Linear regression

The baseline imaging values of the studied cohort can be found in Supplementary Material Table S1. Results of the linear regression between demographics, lesion markers and conventional whole brain MRI features and cognition are reported in Table 2. Demographics alone explained *R*^2^ = 0.29 in both processing speed and memory in our dataset. Whole brain markers explained additional *R*^2^ = 0.06 and *R*^2^ = 0.03 in processing speed and memory, respectively. Next, the addition of MD for processing speed and FA for memory resulted in *R*^2^ values equal to 0.43 and 0.33, respectively.

**Table 2:** The *R*-squared (*R*^2^) and the mean absolute error (MAE) obtained with linear regression of processing speed and memory performance using (i) demographics only, (ii) demographics and conventional lesion and neurodegenerative markers, (iii) model ii + white matter metrics (mean diffusivity, fractional anisotropy, peak-skeletonised mean diffusivity)

*N* = 102	Processing speed			Memory		
	Beta	*p*-value	*R*^2^ [MAE]	Beta	*p*-value	*R*^2^ [MAE]
Model 1: demographics only: age, sex, education						
Age	**−** **0.37**	**< 0.001**	0.29 [0.62]	**−** **0.51**	**< 0.001**	0.29 [0.67]
Sex [male]	**−** 0.02	0.80		**−** 0.11	0.21	
Education	**0.34**	**< 0.001**		**0.17**	**0.05**	
Model 2: model 1 + lesion and atrophy markers						
Age	**−** 0.19	0.07	0.35 [0.61]	**−** **0.38**	**< 0.01**	0.32 [0.65]
Sex [male]	0.03	0.68		**−** 0.07	0.46	
Education	**0.31**	**< 0.001**		0.14	0.11	
Presence of infarcts	**−** 0.05	0.50		**−** 0.02	**−** 0.79	
Presence of micro-bleeds	**−** 0.06	0.48		0.11	0.22	
BPF [%]	**0.29**	**< 0.01**		0.20	0.09	
WMH [%]	**−** 0.04	0.65		0	0.98	
Model 3a: model 2 + average mean diffusivity in white matter						
Age	**−** 0.12	0.23	0.43 [0.55]	**−** **0.35**	**< 0.01**	0.33 [0.65]
Sex [male]	**−** 0.01	0.91		**−** 0.09	0.35	
Education	**0.35**	**< 0.001**		0.16	0.08	
Presence of infarcts	**−** 0.04	0.57		**−** 0.02	0.83	
Presence of microbleeds	-0.03	0.74		0.12	0.17	
BPF [%]	0.16	0.13		0.15	0.24	
WMH [%]	0.04	0.60		0.03	0.73	
MD [mm^2^/s]	**−** **0.34**	**< 0.001**		**−** 0.14	0.19	
Model 3b: model 2 + average fractional anisotropy in white matter						
Age	**−** 0.18	0.07	0.36 [0.59]	**−** **0.38**	**0.001**	0.34 [0.65]
Sex	0.05	0.54		**−** 0.05	0.59	
Education	**0.29**	**< 0.001**		0.13	0.16	
Presence of infarcts	**−** 0.06	0.48		**−** 0.03	0.77	
Presence of micro-bleeds	**−** 0.05	0.52		0.11	0.19	
BPF	**0.27**	**0.01**		0.18	0.13	
WMH [%]	0.03	0.78		0.07	0.51	
FA	0.14	0.16		0.15	0.17	
Model 3c: model 2 + peak-skeletonized mean diffusivity in white matter						
Age	**−** **0.21**	**< 0.05**	0.37 [0.59]	**−** **0.39**	**0.001**	0.32 [0.65]
Sex	0.04	0.59		**−** 0.07	0.48	
Education	**0.31**	**< 0.001**		0.14	0.11	
Presence of infarcts	**−** 0.07	0.41		**−** 0.03	0.77	
Presence of micro-bleeds	**−** 0.04	0.57		0.11	0.21	
BPF [%]	0.18	0.14		0.17	0.21	
WMH [%]	0.11	0.37		0.03	0.82	
PSMD [mm^2^/s]	**−** 0.23	0.10		**−** 0.05	0.74	

For each regressor, we report its normalized regression coefficient (Beta), *p*-value

### Benchmark: linear prediction

Table 3 summarizes the results of the leave-one-out prediction of cognitive performance using conventional metrics, as well as with the addition of the average MD of three specific WM tracts that were previously suggested to be strategic in cSVD. The combination of the whole brain MD with lesion markers, BPF and demographics resulted in the best performance at predicting processing speed (*R*^2^ = 0.38), with an increase of 0.11 and 0.08 in *R*^2^ as compared to the use of models 1 and 2, respectively, and a decrease of MAE equal to 0.06. Conversely, no improvement in the prediction of memory performance was observed for any of the models as compared to the use of demographics only. Next, we evaluated whether tract-based metrics could be beneficial to predict cognitive performance using linear models. The results reported in supporting information Table S3 indicate that tract-based metrics did not improve the performance of linear regression as compared to whole brain metrics in our dataset.

**Table 3:** The mean absolute error (MAE) and *R*-squared (*R*^2^) obtained with linear predictions with leave-one-out validation. Conventional whole brain metrics and established tract-specific metrics were used to predict processing speed and memory scores. Bold indicates the best prediction for each cognitive domain

*N* = 102 subjects		Processing speed		Memory	
Model	Predictors	MAE	*R*^2^	MAE	*R*^2^
Conventional whole brain metrics					
1	Age + sex + education	0.63	0.27	**0.68**	**0.26**
2	1 + lesion markers + BPF	0.63	0.30	0.70	0.25
3a	2 + MD	**0.57**	**0.38**	0.70	0.25
3b	2 + FA	0.63	0.31	0.69	0.25
3c	2 + PSMD	0.62	0.29	**0.69**	**0.26**

### Tract-based ANN prediction

#### ANN features selection

**Fig. 2:**
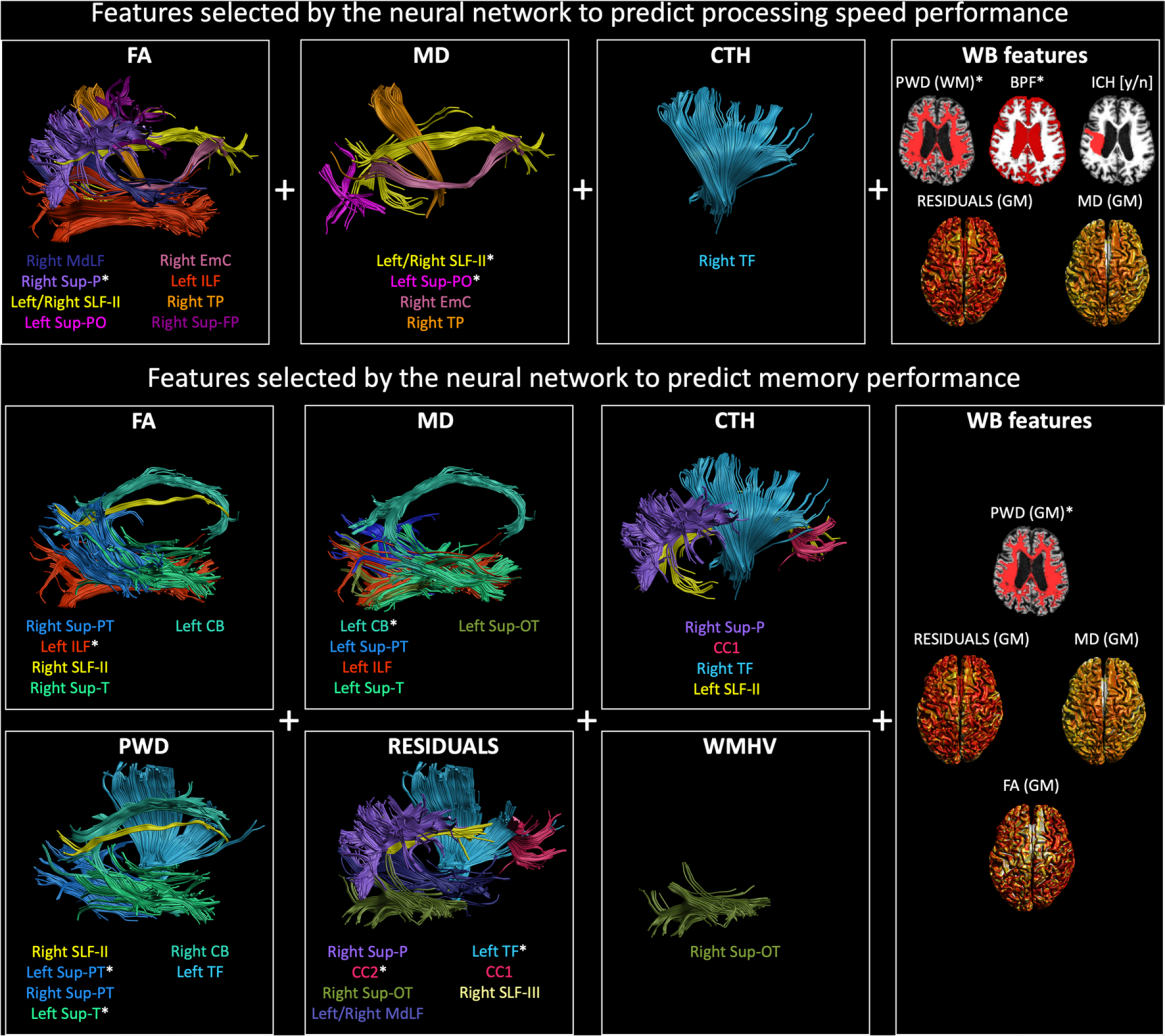
A visual representation of all fiber tracts selected by the 10-iterations artificial neural network (ANN) feature selection procedure on random subsets of 50% of the subjects. The white asterisk shows the features that resulted in the best prediction performance (*R*^2^) in the training set together with age and education as predictors

**Fig. 3:**
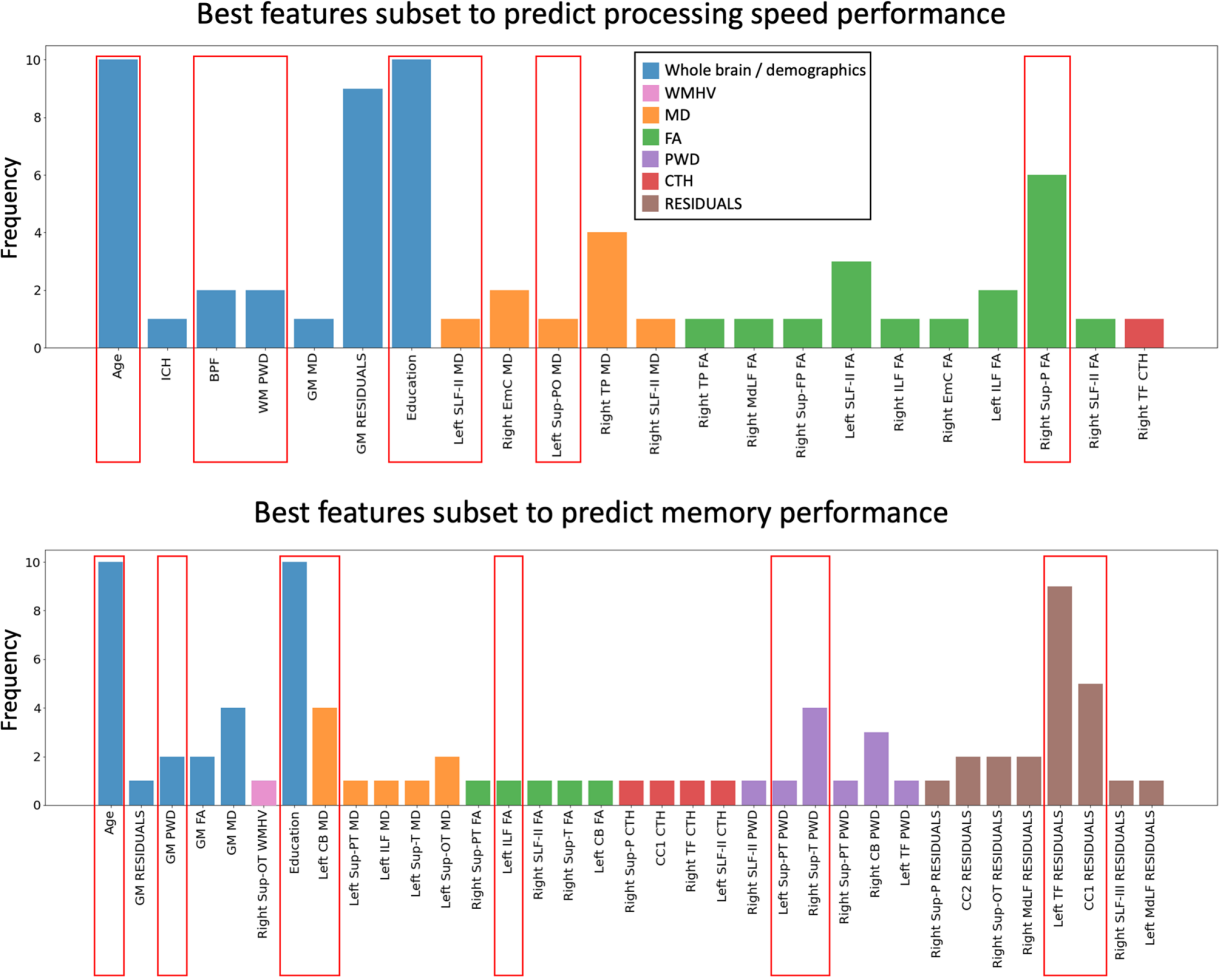
Depicted are all the predictors selected by the artificial neural network (ANN) feature selection on random subsets of 50% of the subjects after 10 iterations for the prediction of processing speed (top) and memory performance (bottom). The red boxes highlight the combination of predictors selected from the ANN in 1 of the 10 feature selection iterations that achieved the best prediction performance in the training set

The results of the features selection procedure on a random selection of 50% of the data to identify the most promising predictors of processing speed and memory performance are shown in Fig. 2. Among all features of the 18 bi-lateral WM tracts relevant for the leave-one-out linear prediction of processing speed in these 51 subjects (Supplementary Material Figure S2), the following features were selected after 10 iterations of feature selection: the average FA of 9 WM tracts, the average MD of 5 WM tracts, the CTH of the cortex connected from the right thalamic-frontal radiation, and 5 whole brain measures. For the prediction of memory performance, a larger number of features was sampled from 15 candidate tracts (Suppl. Fig. S2) as compared to processing speed. These included the FA of 5 WM tracts, the MD of 5 WM tracts, 4 average CTH values, the PWD of 6 tracts, the average residuals of 7 tracts and the WMH volume of the right superior temporo-occipital tract, in addition to 4 whole brain metrics. The frequency with which these features were selected across iterations largely varied, as shown in Fig. 3, with only a minority being repeatedly selected whereas the majority was selected only in a specific subset iteration. Of the 10 iterations, the one providing the highest prediction *R*^2^ in the training set (50% of the subjects randomly selected) is highlighted with white asterisks in Fig. 2 and with red boxes in Fig. 3.

#### ANN prediction evaluation

**Fig. 4:**
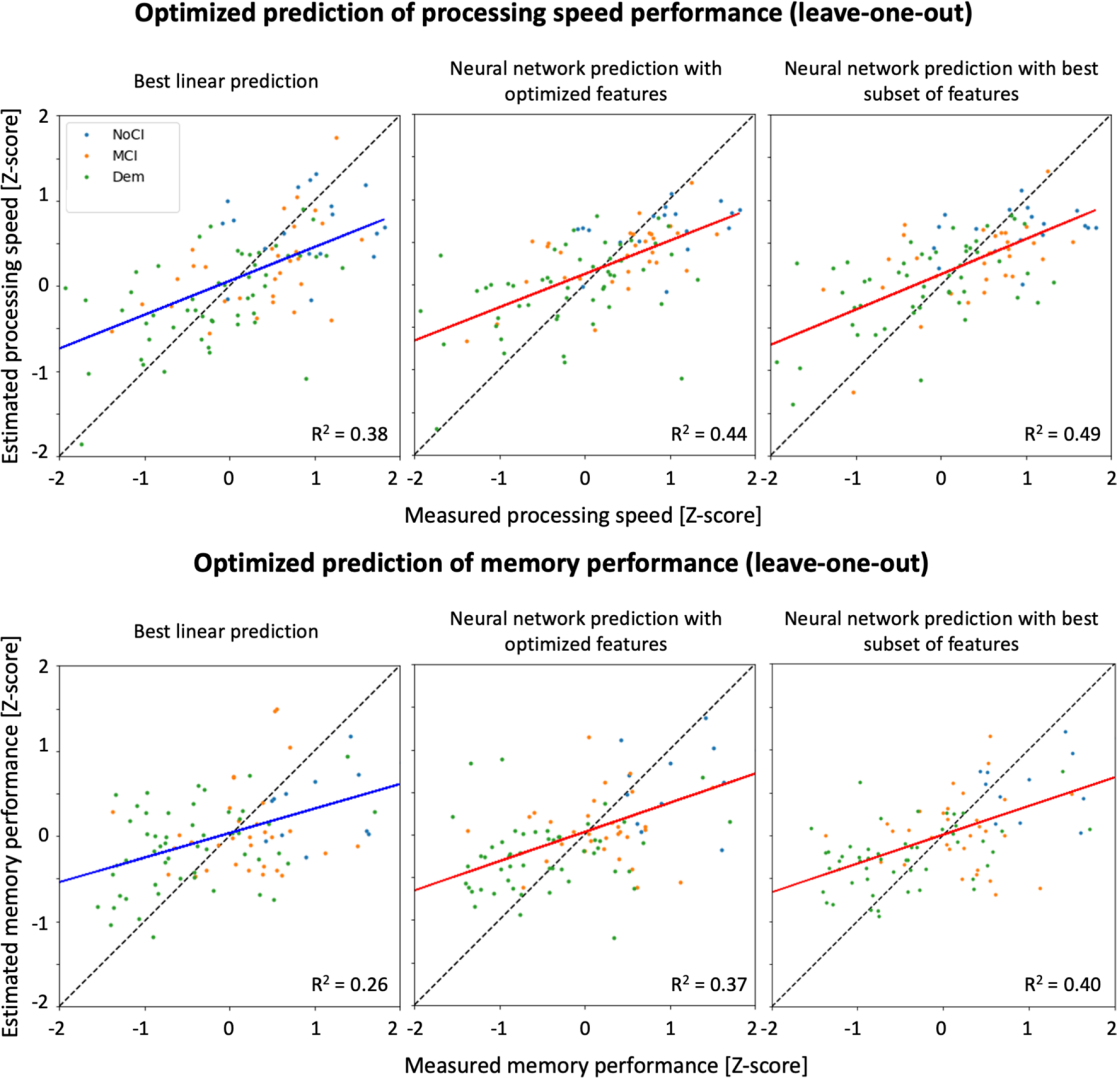
Scatter plots of measured and estimated processing speed (top) and memory performance (bottom) using the linear multivariate predictor (first column) and ANN (second and third column) with leave-one-out cross-validation. The solid line is the regression line, and is colored in blue for multivariate prediction (left), and in red for ANN prediction (middle and right). The colored dots represent each included patient and are colored encoded according to the clinical diagnosis: blue for no cognitive impairment (NoCI), orange for mild cognitive impairment (MCI), and green for patients with dementia (Dem). The best multivariate prediction (left) included demographics, lesion and atrophy markers and average MD in WM, and is compared to predictions with the neural network using all candidate metrics (middle), and the best subset (right)

On the whole dataset, we predicted processing speed and memory performance with both linear regression and ANN as reported in Fig. 4. For ANN, we used both all selected predictors shown in Fig. 2, as well as the best performing subset in the training set. For processing speed, the ANN predictions resulted in *R*^2^ values equal to 0.44 with all candidate predictors, and 0.49 with the best subset, respectively, compared to 0.38 of the best linear regression (whole brain MD + lesion markers + demographics Similarly, the ANN predictions of processing speed achieved the lowest MAE, 0.544 and 0.536, respectively, as compared to 0.566 for the linear regression). The ANN prediction with the best subset of predictors significantly improved the prediction performance as compared to the best linear regression (*F* = 10.32), whereas the improvement observed with all predictors was not significant (*F* = 0.49)—likely penalized by the larger number of predictors. For the prediction of memory, the ANN with all features and with the best subset resulted in *R*^2^ values equal to 0.37 (MAE = 0.619) and 0.40 (MAE = 0.615), respectively, as compared to *R*^2^ = 0.26 (MAE = 0.681) for the best linear model (demographics only). Similarly to what was observed for processing speed, the ANN prediction with the best subset of predictors outperformed the best linear model (*F* = 4.62), whereas the improvement obtained with all candidate predictors was not significant (*F* = 0.41).

In Supporting Information Figure S3, we repeated the ANN prediction using a leave-5-out cross-validation scheme, observing minor changes of prediction performance in terms of *R*^2^ (reductions up to 0.02).

## Discussion

We have shown proof-of-concept that integrating tract-specific multimodal MRI metrics with an artificial neural network framework can outperform conventional methods at predicting cognitive performance in memory clinic patients with small vessel disease. Compared to the best linear predictors selected in this work, the optimized ANN framework explained additional *R*^2^ = 0.09 predicting processing speed and *R*^2^ = 0.14 when predicting memory performance, and thus represents a promising framework toward a better characterization of cognitive performance based on concurrent MRI.

### Multimodal imaging better explains cognitive performance than individual metrics

An important result of our work is to confirm that the integration of multiple modalities, such as dMRI and sMRI (T1-weighted, FLAIR), is needed to achieve a better understanding of SVD-related brain injury and of its impact on brain function. Table 2 unequivocally shows that combining established DTI metrics—even at the whole brain level—with lesion markers and non-imaging information (e.g., demographics) better captures inter-subject variation in cognitive performance in this clinically heterogeneous study cohort, which is in line with previous observations from our group and others (Baykara et al. 2016; Biesbroek et al. 2018; Duering et al. 2018; Groeneveld et al. 2019; Boomsma et al. 2020; de Lange et al. 2020; Jokinen et al. 2020). In our study, we have chosen to consider demographics as part of the model rather than regressing their effect out from the data as part of a multi-step regression approach. While this increases the complexity of the considered model, it allows to consider collinearities between predictors that could otherwise potentially lead to biases, as previously shown in other fields (Freckleton 2002). Interestingly, the inclusion of imaging markers increases the amount of explained variance (e.g., group level) during regression (Table 2), but not in leave-one-out prediction of individual memory scores (Table 3). A possible explanation for this observation is the existence of a weak (linear) relation between predictors and outcome, which is difficult to estimate and substantially changes even when excluding a single measurement, collinearity between predictors, or the existence of non-linear relations between (some) predictors and cognitive performance that cannot be captured with linear prediction.

### ANN prediction methods

Considering multimodal tract-specific metrics proved advantageous to predict the considered cognitive domains only in combination with a feed-forward artificial network. This finding well aligns with recent literature showing the need for methods beyond linear models when dealing with many imaging predictors, to account for their collinearity and, potentially, for their non-linear relation with the outcome. Examples of these methods include variance decomposition algorithms, such as principal component analysis which have previously been used to combine tract-specific metrics (Chamberland et al. 2019), and methods based on with sparsity constraints (Schouten et al. 2017; Boot et al. 2020; Cole 2020). In the latest years, ANN have emerged not only as a versatile tool to achieve image segmentation and other tasks both in research and clinical practice (van Rijn and De Luca 2020), but also to perform prediction by learning complex relations between multiple input metrics and outcome while potentially dealing with collinearity. In this work, we have shown that ANN can explain additional *R*^2^ values up to 0.10–0.13 when predicting processing speed and memory performance, respectively, as compared to conventional methods. Importantly, using the best predictors from the ANN feature selection as input to a linear model slightly improved the prediction of processing speed from *R*^2^ = 0.38 to 0.39, and of memory from *R*^2^ = 0.26 to 0.33, which is well below what is observed with the corresponding ANNs (*R*^2^ = 0.49 and *R*^2^ = 0.40, respectively). Overall, this suggests that most of the gain in performance of the ANN prediction is driven by the ability of ANNs to handle eventual collinearities between predictors and to account for eventual non-linear relations with outcome as compared to conventional linear methods. Of note, other methods to account for these effects can be found in machine learning literature (e.g., support vector machines, nonlinear principal component analysis and regression, random forests, etc.), and might prove equally advantageous to ANNs to overcome limitations of linear approaches. Demonstrating which of these methods is the most advantageous to relate multimodal tract-based metrics to cognition remains an open question for future work.

### ANN feature selection

We have introduced a feature selection strategy to support the performance of the ANN with a relatively small sample size, which proved key to the final performance of the method. In studies with larger samples, which are becoming easier to achieve, thanks to the ability to pool multi-site that with data harmonization methods (de Brito Robalo et al. 2021; de Luca and Biessels 2021), this step might be less relevant. In that case, deeper networks (i.e., with more hidden layers) might be able to prove the identity of relevant predictive features without further tweaking. In most neuroimaging studies, however, achieving large sample sizes remains challenging, and our feature selection strategy might prove promising to the success of ANNs in this context, especially to prevent overfitting and their consequent poor generalization in unseen subjects. Nevertheless, it should be reminded that ANNs depend on random initialization factors and hyper-parameters choices, and their training might therefore not always converge to a global minimum especially with limited sample sizes as those employed in this study. Taking these factors into account, it is likely that running the feature selection procedure de novo would result in a different—only partly overlapping selection of features, which suggests the need for great care when attempting biological interpretations. In future studies, fixing the randomization seed and discarding non-deterministic components during the design of ANN architectures could prove advantageous to support interpretation and reproducibility. Independently from these technical solutions, the use of larger datasets than the one in this study should intrinsically lead to more consistent feature selections, allowing the ANN to train more extensively and be thus likely less prone to initialization parameters and local minima.

### Comparison with previous studies on cognition in SVD

There is increasing awareness in the field of vascular cognitive impairment that brain lesions observed on T1-weighted imaging and FLAIR only represent the tip of the iceberg of the ongoing pathological processes, and that dMRI metrics might better capture “hidden” brain injury and its relation to cognitive performance. These considerations hold also in our result, as dMRI metrics outperformed other imaging markers at both whole brain and at tract-specific level. Previous studies in SVD and beyond have suggested that the location of brain lesions (e.g., WMH) is predictive of their impact on specific cognitive functions through mechanisms such as brain disconnection. Indeed, the potential of shifting the analysis focus to the tract level to better capture cognitive function is supported by a growing body of evidence suggesting the importance of understanding lesions in the context of brain connections (Fox 2018), and the existence of a direct link between specific white matter tracts and brain function (Thiebaut de Schotten et al. 2020), also in cSVD (Biesbroek et al. 2018). Following a similar concept, the MetaVCI-Map (Biesbroek et al. 2017; Weaver et al. 2019, 2021) study has recently showcased the idea of predicting the impact of lesions on brain cognition based on their location within the brain white matter, although no localization of the tracts with dMRI was involved. Interestingly, in our analyses WMH burden was not selected by our feature selection procedure neither at whole brain nor at tract-specific level to predict processing speed. When we performed a linear regression using the amount of WMH of each WM tract as predictor (Supporting Information Table S3), we obtained a worse prediction of both processing speed and memory than when using demographics only, suggesting a tendency toward overfitting of this metrics and perhaps a lack of specificity to the different etiologies included in our study sample. Altogether, this might indicate that although WMH of specific tracts are related to brain function at the group level, they do not generalize to prediction tasks, such as the prediction of individualized cognitive function.” Regarding other lesion markers, we should note that we only considered the presence/absence of lacunes and micro-bleeds, which did not allow us to evaluate their effect at the tract level.

While this study only represents a proof-of-concept of the potential of the proposed framework, it is interesting to note that the selected features well agree with previous literature in cSVD. For example, features predictive of processing speed performance (Fig. 2 and Table 3) include both markers of neurodegeneration (BPF), of global WM injury (PSMD), and 3 tracts that have been previously related to processing speed tasks (Turken et al. 2008; Sasson et al. 2013), such as the superior longitudinal fasciculus and 2 superior parietal tracts. For the prediction of memory, only metrics sampled in the whole GM were selected in addition to several metrics of tracts previously suggested to be involved in memory tasks, including the cingulum, the forceps minor, occipital tracts, and the thalamic projections. Of note, our considered features did not include the hippocampal volume, which is commonly used to predict memory performance and might be worth considering in future studies.

### Limitations and strengths

#### Overfitting

This is one of the major risks when investigating prediction methods in modest sample sizes, especially when based on machine learning and ANN. Our prediction framework was designed with awareness to this potential issue but is not free of limitations. First, the number of total predictors was reduced to a more viable subset by means of a feature selection strategy which was run on a random selection of 50% of the subjects, and was thus not optimized for the whole dataset. Considering our limited sample size, we opted for leave-one-out cross-validation for both linear prediction and ANN, to retain as many training subjects as possible while validating the prediction on unseen subjects. To further evaluate the generalizability of the proposed tract-based ANN framework, we have repeated the prediction using a leave-5-out cross-validation scheme observing minor reductions in prediction performance (Supporting Information Figure S3), which supports a limited impact of overfitting on our results. Nevertheless, this validation approach might still underestimate the effective generalizability of the method. For example, the transformation of input metrics to *Z*-scores was performed once for all data points—including those that become part of leave-one-out (or leave-5-out validation), which might be prone to leakage of information from the training to the validation set. For this reason, future validation of this framework in a (large) external cohort remains needed before attempting a biological interpretation of the findings.

#### Optimization

Another aspect of ANN that can strongly influence their performance is the choice of hyper-parameters (Isensee et al. 2021), including the network architecture and the optimization settings. In this study, we empirically opted for a shallow network with 2 layers and a limited number of nodes (20) to minimize the chance of overfitting given our limited training set. Hence, the architecture used in this work does not represent an optimal configuration for all applications but rather a starting point to further optimize in each specific application, and further research is required to determine objective rules to guide the choice of hyper-parameters. Conventional techniques as the linear regression require less user choices but are still prone to overfitting. This is shown, for example, by the fact that adding tract-specific metrics can lead to worse performance than just using demographics (Supporting Information Table S3). In this work, we have chosen to allow up to 10 predictors in the linear regression. This is an arbitrary choice that mediates between the risk of under-fitting and overfitting. Of note, several methods can be found in literature to improve the performance of a linear regression, including principal component analysis, but their extensive implementation is beyond the scope of this work, and impacts the ability to interpret which metrics are actually relevant to the prediction.

#### MRI data

We have shown proof-of-concept that integrating metrics from different commonly acquired imaging modalities can substantially improve our ability to predict cognitive performance. Nevertheless, the diffusion protocol here included did not allow to investigate diffusion metrics beyond the diffusion tensor, such as diffusion kurtosis imaging or other advanced models which have been shown superior to DTI in terms of sensitivity to microstructural changes in a number of applications, including SVD (Konieczny et al. 2021). On the same note, multi-shell dMRI protocols with higher diffusion weighting than the one employed in this study would likely allow to further improve the performance of the WM tracts (Jeurissen et al. 2014) reconstruction and of the underlying GM properties (De Luca et al. 2020). Besides dMRI, the inclusion of other imaging modalities, such as arterial spin labeling or cerebrovascular reactivity (van den Brink et al. 2021), would likely be favorable to further characterize a disease like SVD, which is of vascular etiology.

## Conclusion

In conclusion, we have shown that integrating multimodal metrics in a framework based on artificial neural networks is advantageous to predict cognitive performance in a memory clinic setting. Our framework outperforms linear methods at predicting cognitive performance, representing a step forward toward individualized predictions in patients with cerebral small vessel disease.

## Supplementary Information

Below is the link to the electronic supplementary material.

Supplementary file 1 (GIF 177075 KB)

Supplementary file 2 (GIF 238779 KB)

Supplementary file 3 (GIF 223661 KB)

Supplementary file 4 (DOCX 1416 KB)

## Data Availability

The datasets generated during and/or analyzed during the current study are not publicly available because of the involvement of participants of a clinical study for which data publication is not approved. The data are nonetheless available from the corresponding author on reasonable request.
