# Supplementary material for: Multimodal tract-based MRI metrics outperform whole brain markers in determining cognitive impact of small vessel disease-related brain injury
Source: Brain Struct Funct. 2022 Aug 22;227(7):2553–67. doi: 10.1007/s00429-022-02546-2 (PMC9418106; doi:10.1007/s00429-022-02546-2)
Supplement: Supplementary file 4 — (DOCX 1416 KB) [file 429_2022_2546_MOESM4_ESM.docx]

# Supplementary Material


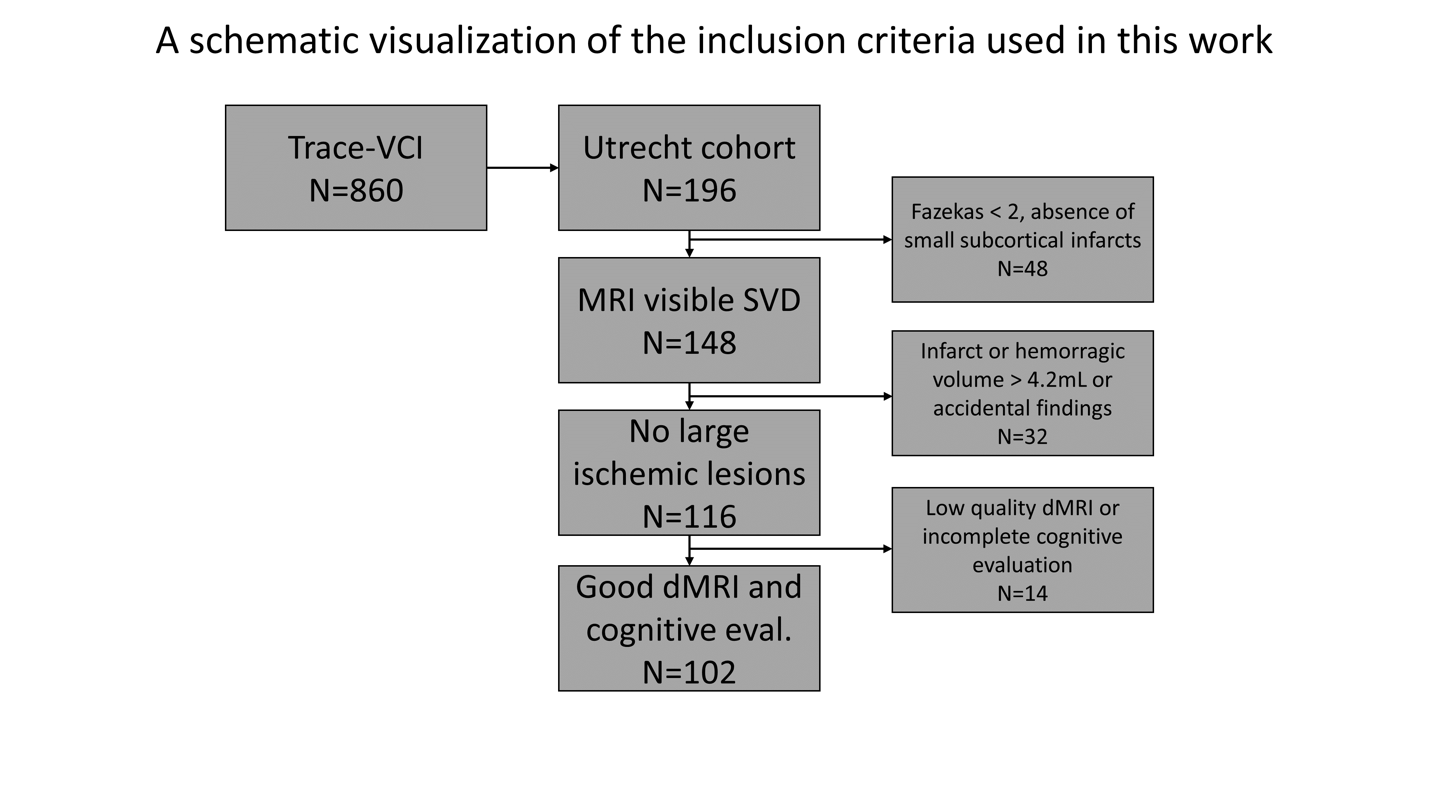


Figure S1: a visualization of the inclusion/exclusion criteria adopted in this work.

| Table S1: A list of the tracts reconstructed by automated clustering method and their abbreviation used in the main text. | |
| --- | --- |
| Abbreviation | Definition |
| AF | arcuate fasciculus |
| CB | cingulum bundle |
| EC | external capsule |
| EmC | extreme capsule |
| ILF | inferior longitudinal fasciculus |
| IoFF | inferior occipito-frontal fasciculus |
| MdLF | middle longitudinal fasciculus |
| PLIC | posterior limb of internal capsule |
| SLF-I-II-III | superior longitudinal fasciculus I-II-III |
| UF | uncinate fasciculus |
| CPC | cortico-ponto-cerebellar |
| ICP | inferior cerebellar peduncle |
| Intra-CBLM-I&P | intracerebellar input and Purkinje tract |
| Intra-CBLM-PaT | intracerebellar parallel tract |
| MCP | middle cerebellar peduncle |
| CC 1-7 | corpus callosum segments 1 to 7 (includes forceps) |
| CST | corticospinal tract |
| CR-F | corona-radiata-frontal |
| CR-P | corona-radiata-parietal |
| SF | striato-frontal |
| SO | striato-occipital |
| SP | striato-parietal |
| TF | thalamo-frontal |
| TO | thalamo-occipital |
| TP | thalamo-parietal |
| Sup-F | superficial-frontal |
| Sup-FP | superficial-frontal-parietal |
| Sup-O | superficial-occipital |
| Sup-OT | superficial-occipital-temporal |
| Sup-P | superficial-parietal |
| Sup-PO | superficial-parietal-occipital |
| Sup-PT | superficial-parietal-temporal |
| Sup-T | superficial-temporal |

| Table S2: average ± standard deviation of lesion and atrophy markers in the considered cohort. | |
| --- | --- |
| Brain atrophy | |
| Brain parenchymal fraction (BPF) [%] | 0.67 ± 0.04 |
| Cortical thickness [mm] | 2.53 ± 0.13 |
| Cerebrovascular lesions | |
| WMH volume [% of ICV] | 3.4% ± 2.5% |
| Non-lacunar (sub)cortical infarcts | 26 (26%) |
| Lacunar infarcts | 50 (49%) |
| Microinfarcts | 4 (4%) |
| Intracerebral hemorrhages | 7 (7%) |
| Any microbleeds | 42 (41%) |
| Microstructural alterations | |
| Mean diffusivity of WM [mm^2^/s] | (0.88 ± 0.05) x 10^-3^ |
| Mean diffusivity of GM [mm^2^/s] | (1.07 ± 0.07) x 10^-3^ |
| Peak-width mean diffusivity of WM [s/mm^2^/s] | (1.10 ± 0.35) x 10^-3^ |
| Peak-width mean diffusivity of GM [s/mm^2^/s] | (1.80 ± 0.30) x 10^-3^ |
| Fractional anisotropy of WM | 0.28 ± 0.02 |
| Fractional anisotropy of GM | 0.13 ± 0.01 |

| Table S3: Linear multivariate prediction (leave one out) using tract-specific imaging metrics. Bold indicates the best prediction for each domain | | | | | | | |
| --- | --- | --- | --- | --- | --- | --- | --- |
| **N=102 subjects** | | **Processing speed** | | | **Memory** | | |
| **Model** | **Predictors** | **MAE** | **R^2^** | **Relevant tracts** | **MAE** | **R^2^** | **Relevant tracts** |
| 1 | Age + Sex + Education + WMH burden | 0,70 | 0,16 | Right CB  CC  Left CR-F  Left CST  Left/Right IOFF  Right MdLF  Right SF  Right SP  Right Sup-F  Right Sup-OT  Right Sup-PT  Left/Right TF | 0,78 | 0,12 | Left/Right CB  CC  Left/Right CR-F  Right CST  Right ILF  Right Sup-OT  Right Sup-PT  Right Sup-T  Left/Right TF |
| 2 | Age + Sex + Education + MD | 0,63 | 0,27 | Left/Right CB  Left/Right CR-F  Left/Right CST  Right ILF  Left/Right SF  Left/Right SP  Left/Right Sup-P  Left/Right Sup-T  Right TF | **0,69** | **0,31** | **Left/Right CB**  **CC**  **Left/Right ILF**  **Left MdLF**  **Left/Right Sup-OT**  **Left Sup-PT**  **Left/Right Sup-P**  **Left/Right Sup-T**  **Left/Right UF** |
| 3 | Age + Sex + Education + FA | **0,62** | **0,30** | **Right CR-F**  **Left CST**  **Left/Right EmC**  **Left/Right ILF**  **Right ILF**  **Left MdLF**  **Right SLF-III**  **Left SLF-II**  **Left/Right Sup-FP**  **Left/Right Sup-PO**  **Left/Right Sup-P**  **Right TF**  **Right TP** | 0,70 | 0,22 | Left EmC  Left Sup-PO  Right Sup-P  Left UF |
| 4 | Age + Sex + Education + PWD | 0,67 | 0,21 | CC  Left/Right CR-F  Left/Right ILF  Right MdLF  Right SF  Left SP  Right Sup-OT  Left/Right Sup-PT  Right Sup-P  Left/Right Sup-T  Left TP | 0,70 | 0,28 | Left CB  Right CR-F  Right EmC  Left/Right ILF  Right IOFF  Right MdLF  Right SF  Right Sup-OT  Right Sup-PT  Left/Right Sup-T  Left TP  Left/Right UF |
| 5 | Age + Sex + Education + Residuals | 0,63 | 0,25 | Right EmC | 0,67 | 0,28 | Right CST  Left/Right EmC  Left/Right MdLF |
| 6 | Age + Sex + Education + CTH | 0,71 | 0,11 | CC  Right CR-F  Right CST  Left CBLM-I&P  Right SLF-III  Left SLF-II  Left TP | 0,75 | 0,18 | CC  Right CST  Left EmC  Left Sup-FP  Right Sup-F  Left Sup-PT  Left/Right Sup-T  Left/Right TP  Right UF |


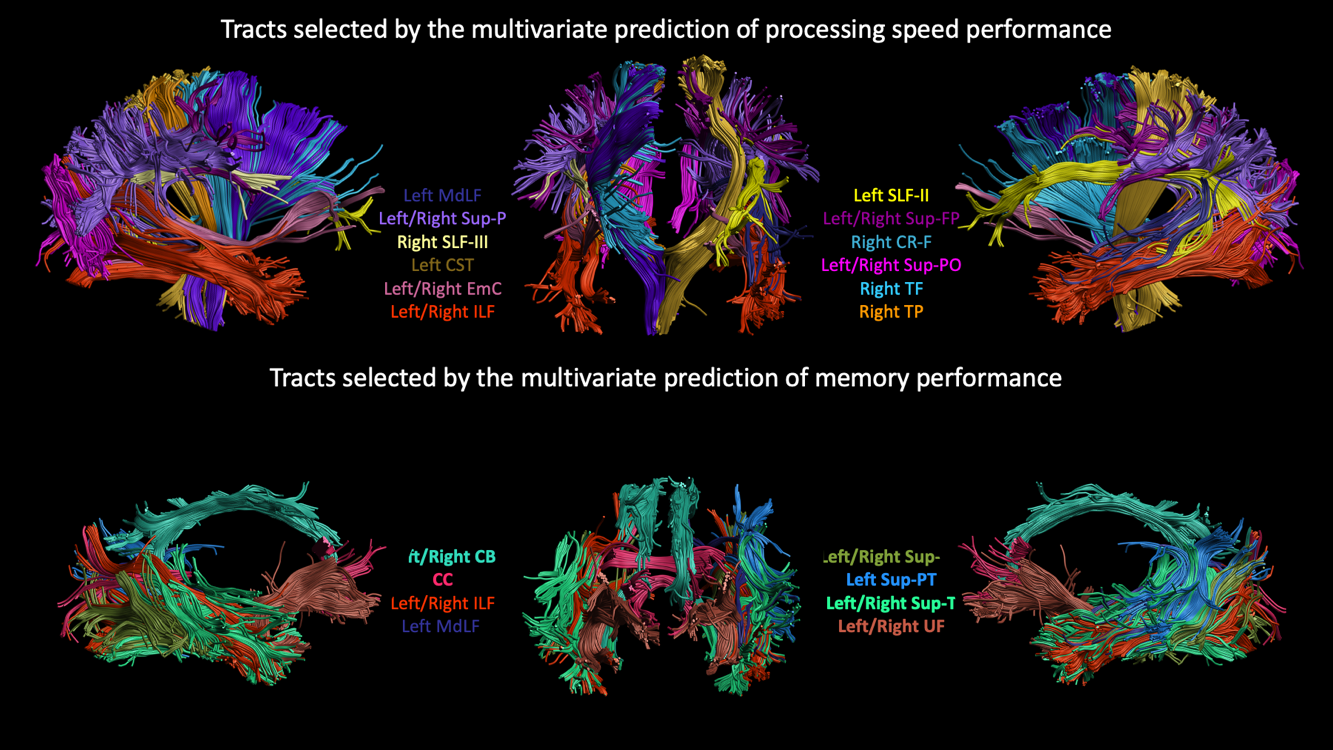


Figure S2: The tracts selected by the multivariate regression that best predicted processing speed (top) and memory performance (bottom) with leave-one-out cross validation.


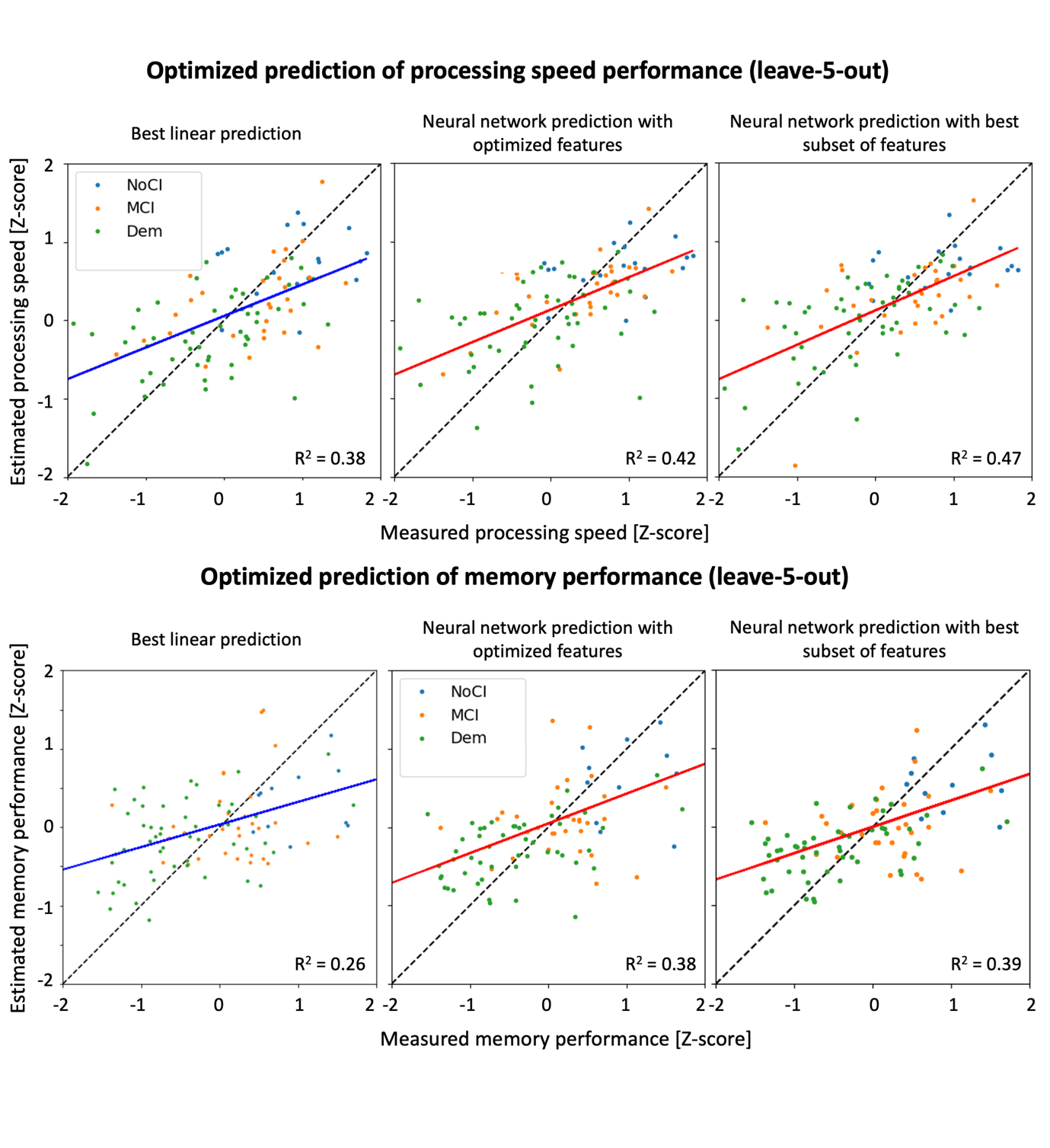


Figure S3: Scatter plots of measured and estimated processing speed (top) and memory performance (bottom) using the linear multivariate predictor (first column) and ANN (second and third column) with leave-5-out cross-validation. The solid line is the regression line, and is colored in blue for multivariate prediction (left), and in red for ANN prediction (middle and right). The colored dots represent each included patient and are colored encoded according to the clinical diagnosis: blue for no cognitive impairment (NoCI), orange for mild cognitive impairment (MCI), and green for patients with dementia (Dem). The best multivariate prediction (left) included demographics, lesion and atrophy markers and average MD in WM, and is compared to predictions with the neural network using all candidate metrics (middle), and the best subset (right).

Video S1: Subsequent axial slices (feet-head) of the spatial probability of each reconstructed white matter tract at the group level.

Video S2: Subsequent coronal slices (fronto-occipital) of the spatial probability of each reconstructed white matter tract at the group level.

Video S3: Subsequent sagittal slices (right-left) of the spatial probability of each reconstructed white matter tract at the group level.
